# Epidemiological, Clinical, and Histopathological Characteristics of Patients with Sporotrichoid Lymphocutaneous Infection in Southern Thailand: A 10-Year Retrospective Study

**DOI:** 10.4269/ajtmh.22-0300

**Published:** 2023-02-13

**Authors:** Tanapong Wongrat, Kumpol Aiempanakit, Siripen Kanchanasuwan

**Affiliations:** Department of Internal Medicine, Faculty of Medicine, Prince of Songkla University, Songkhla, Thailand

## Abstract

Sporotrichoid lymphocutaneous infection is caused by a variety of pathogens. However, in most cases, the causative pathogen cannot be identified on the basis of clinical and histopathological features. We examined the clinical manifestations, histopathologic findings, causative pathogens, treatment, and prognostic factors of sporotrichoid lymphocutaneous infection, specifically in the context of Thailand. The electronic medical records of patients with sporotrichoid lymphocutaneous infection who visited Songklanagarind Hospital from January 2000 to December 2010 were reviewed. A total of 53 patients were included; 41 (77.4%) were female, 12 (22.6%) were male, and the mean (SD) age was 52.9 (± 15.9) years. Nodules, plaques, and papules were the most commonly observed morphologies. Upper extremities were the most commonly infected sites. Mammal-caused injuries were associated with fungal infection but not at a statistically significant level. The most common histopathologic finding was suppurative granuloma. The identified causative pathogens were mainly dematiaceous fungus and occasionally nontuberculous *Mycobacterium*. Itraconazole was the medication of choice for empiric and specific treatment of the patients with confirmed fungal infection. Dematiaceous fungi were the most common identified pathogens causing sporotrichoid lymphocutaneous infection in southern Thailand. Empirical itraconazole is useful, especially in patients who report contact injury caused by pets at the primary lesion site. Skin biopsy for tissue histopathology and culture is essential.

## INTRODUCTION

Sporotrichoid lymphocutaneous infection is characterized by granulomatous skin lesions that progress linearly along the lymphatic tract.^1^ It is a distinctive but often unrecognized syndrome,^2^ with lesions commonly appearing on upper and lower extremities.^3^ This syndrome is caused by various organisms, including fungi, mycobacteria, bacteria, viruses, and parasites.^4^^–^^10^ *Sporothrix* spp., a fungus that occurs worldwide, has been recognized as a common pathogen causing sporotrichoid lymphocutaneous infection.^11^ Increasing rates of the infection caused by *Mycobacterium*^12^^,^^13^ and dematiaceous fungus^14^ have been reported recently in Asian countries, especially in Thailand. Although systemic symptoms are rarely observed in the patients, the lesions caused by different species have many similar characteristics: 1) The primary lesion arises from contact with the environment media inhabited by the pathogenic organisms, such as soil, water, environmental niches, and animals, and 2) granulomatous inflammation is usually observed upon pathological examination.

The skin lesions and histopathological findings in cases caused by different pathogens were very similar.^4^^–^^10^^,^^15^ Skin biopsy and culture was considered a standard diagnostic procedure for this diseases, but the yield of identified pathogens was very low. In addition, in skin cultures, *Sporothrix* spp. has low mean colony-forming units and long mean colony growth time.^16^

*Sporothrix* spp. is the most common pathogen globally, whereas nontuberculosis *Mycobacterium* (NTM) is the most common pathogen in Asia,^13^ especially Thailand.^12^ In addition, dematiaceous fungi (e.g., *Fonsecaea* spp., *Exophiala* spp., and *Wangiella* spp.), which usually cause chromoblastomycosis, have been reported to cause sporotrichoid lymphocutaneous syndrome in some cases.^14^ The treatment strategy for lymphocutaneous syndrome is complex. The minimum inhibitory concentration of the antibiotic itraconazole against *S. schenckii* has been increasing, but cases of drug resistance have not been identified.^17^

The presence of many causative organisms, unspecific clinical presentations, similar tissue pathology to other diseases, and long time to identify pathogens lead to delayed diagnosis and inappropriate use of antibiotics, which may have a negative impact on progression of the lesions and increase the complexity of treatment required.

In this study, we investigated the clinical manifestations, histopathological and microbiological features, and treatment of patients with sporotrichoid lymphocutaneous infection using a 10-year data pool of a large tertiary hospital.

## MATERIALS AND METHODS

The present study was a retrospective study. The electronic medical records of patients with sporotrichoid subcutaneous infections who visited Songklanagarind Hospital, a tertiary university-based hospital in southern Thailand, from January 2010 to December 2019 were searched. The study was approved by institutional review board of the Faculty of Medicine, Prince of Songkla University REC: 63-195-14-1. We included patients who presented with skin lesions in a sporotrichoid pattern, as described by physicians (mostly by dermatologists) and whose results of histopathological examination and tissue culture of skin biopsy samples were available. Patients who had not completed the follow-up process were excluded. Data on demographic features, underlying disease, types of injury, clinical manifestations, histopathological findings, microbiological culture results, and treatment regimens were collected. Cure was defined as healed lesion and no sign of clinical activity after treatment cessation for at least 1 month. Recurrence was defined as the presence clinical activity after clinical cure, at the same sites as previously recorded, observed at any follow-up period of 6 months, and treatment failure was defined as no improvement or worsening of the skin lesions after 1 month of therapy.^18^

For statistical analysis, a χ^2^ test was used to compare differences in categorical data, and an independent *t* test was used to compare continuous variables. Statistical significance was set at a *P* value of < 0.05. All analyses were performed using SPSS software (SPSS Inc., Chicago, IL).

## RESULTS

From the 10-year retrospective chart review, we identified 57 patients with nodular lymphangitis. Four patients (7%) were excluded from the study due tor loss to follow-up. Finally, the data of 53 patients were analyzed in this study. The clinical data are provided in Table 1.

**Table 1:** Characteristics of cutaneous infection with sporotrichoid pattern

Variables	Results
Sex	
Male	12 (22.6%)
Female	41 (77.4%)
Age (years)	
Range	16–82
Mean (± SD)	52.5 (15.9)
15–40	11 (20.8%)
4160	22 (41.5%)
> 61	20 (37.7%)
Occupation	
Agriculturist	4 (7.5%)
Fisherman	2 (3.8%)
Student	4 (7.5%)
Office worker	9 (17%)
Shopkeeper	3 (5.7%)
Laborer	7 (13.2%)
Other/no occupation	24 (13.2%)
Duration of disease	
Range	7 days to 3 years
Mean	91.5 days
< 3 months	41 (77.4%)
3–6 months	8 (15.0%)
6–12 months	2 (3.8%)
> 1 year	2 (3.8%)
History of injury	
Present	31 (58.5%)
Absent	22 (41.5%)
Type of injury	
Cat bite	20 (64.5%)
Fish handling	4 (12.9%)
Thorn prick	5 (16.1%)
Insect bite	2 (6.5%)

**Figure 1.**
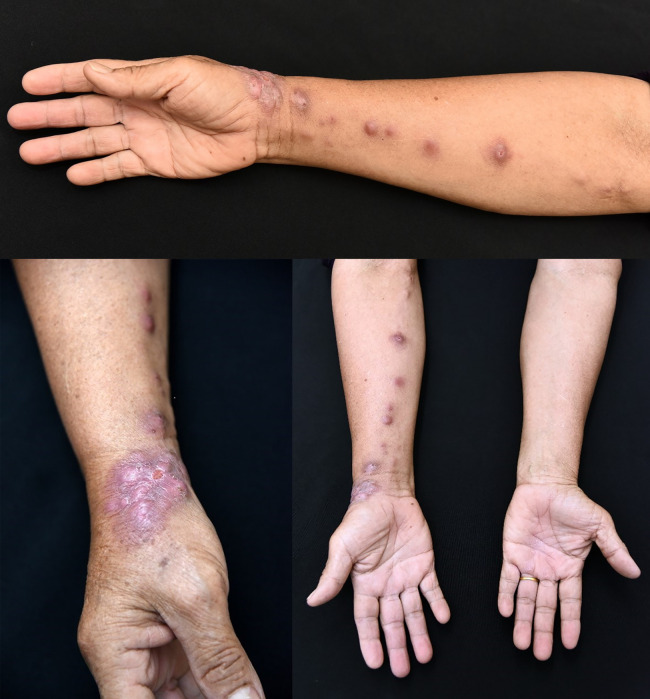
Noduloulcerative plaque on right wrist and subcutaneous erythematous nodules along lymphatic tract on the right forearm.

**Figure 2.**
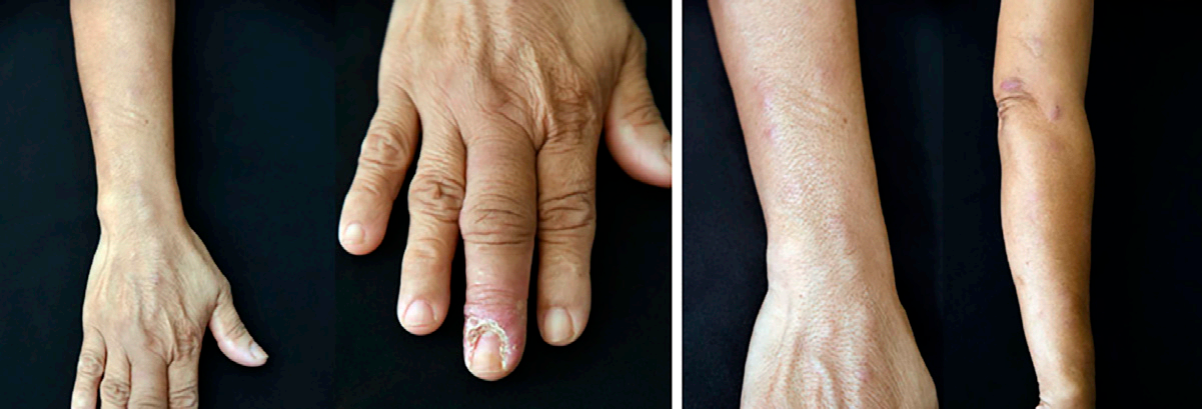
Ulcer at a middle finger and subcutaneous erythematous nodules along lymphatic tract on the right forearm.

The frequency of sites of primary lesions varied (Table 2). The upper extremities were the most common lesion sites (41 patients, 77.4%), followed by lower limbs (eight patients, 15.1%) and head and neck (four patients, 7.5%). The clinical morphologies of the lesions according to the pathogenic organism are shown in Supplemental Table 1. The representative photographs of the different types skin lesions are shown in Figures 1 and 2.

**Table 2:** Skin manifestations and lymph node involvement

Variables	Result		
	Single morphology	Combined morphology	Total
Nodule	10	18	28
Papule	5	10	15
Plaque	10	9	19
Patch	1	0	1
Pustule/abscess	2	6	8
Ulcer	1	9	10
Sites of primary lesions			
Upper extremities	41 (77.4%)		
Lower extremities	8 (15.1%)		
Head and neck	4 (7.5%)		
Lymphadenopathy			
Present	10 (18.9%)		
Absent	43 (81.1%)		

The major histopathological finding (Supplemental Table 2) was granulomatous inflammation (45 patients, 84.9%), including suppurative granuloma (16 patients, 30.2%), tuberculoid granuloma (five patients, 9.4%), mixed granuloma (one patient, 1.9%), and unspecified granuloma (23 patients, 43.3%).

Among the 53 skin biopsy samples (Supplemental Table 3), positive culture was found in 24 samples (45.3%). The major causative agents were fungi, including *Exophila* spp. (seven patients, 29.1%), *Wangiella* spp. (six patients, 25.0%), *Cladorrhinum bulbillosum*, (one patient), *Rhinocladiella* spp. (one patient), *Fonsecace* spp. (one patient), unidentified black mold (two patients), and *Sporothrix* spp. (one patient). NTM was identified in five patients (21.7%). Special stains, including Gomori methenamine silver, periodic acid-Schiff, and acid-fast bacillus stains, were performed in both positive and negative culture samples. However, our study found that these special stains failed to identify any pathogen.

### Treatment.

Twenty-seven patients (50.9%) were treated with antimicrobial medications before visiting our hospital. Empirical treatment was defined as the treatment that was initiated once the patients were clinically diagnosed with sporotrichoid lymphocutaneous syndrome and waiting for skin biopsy and culture reports. The most frequently prescribed medications were itraconazole (30 patients, 56.6%) and antituberculous agents (six patients, 11.3%). Other regimens included monotherapy with doxycycline, dicloxacillin, or others and combination therapy with ciprofloxacin plus clarithromycin, doxycycline plus clarithromycin, or others. Twenty patients (69%) of those with negative cultures in this study were treated with itraconazzole; the remaining patients received other antibiotic regimens, including monotherapy or combination therapy of clarithromycin, ciprofloxacin, doxycycline, and others. All of the patients in this study had clinical cure after treatment.

### Factors associated with culture-positive result and fungal infection.

Only history of mammal-caused injury was associated with positive fungal culture, but not at a statistically significant level (*P* = 0.057) (Table 3).

**Table 3:** Factors associated with positive culture and fungal infection

Factors	Positive culture (fungi and NTM), *N* (%)	Negative culture, *N* (%)	OR (95% CI)	*P* value
History of injury			0.384 (0.10–1.37)	0.097
Report injury	7 (13.21)	15 (28.30)		
Nonreported injury	17 (32.08)	14 (26.42)		
Lymphadenopathy			0.766 (0.13–3.79)	0.709
Positive lymphadenopathy	4 (7.55)	6 (11.32)		
Negative lymphadenopathy	20 (37.74)	23 (43.40)		
Treatment before skin biopsy			1.5 (0.44–5.14)	0.465
Received treatment	14 (26.42)	14 (26.42)		
Did not receive treatment	10 (18.87)	15 (28.30)		

	Positive fungal culture	Negative fungal culture		
Mammal-related injury			3.086 (0.81–11.78)	0.057
Reported injury	10 (18.87)	9 (16.98)		
Nonreported injury	9 (16.98)	25 (47.17)		
Skin morphology			1.74 (0.42–6.35)	0.336
Plaques	11 (20.75)	15 (28.3)		
Ulcers	8 (15.09)	19 (35.85)		

NTM = nontuberculosis *Mycobacterium*; OR = odds ratio.

## DISCUSSION

To our knowledge, this is the first study on the epidemiology of sporotrichoid lymphocutaneous infection in Thailand. Sporotrichoid lymphocutaneous syndrome is an uncommon disease that is rarely recognized by healthcare professions. The classic manifestation is sporotrichosis, but this could be caused by various pathogens in different habitats, requiring accurate treatments. In previous studies, sporotrichosis, leishmaniasis, nocardiosis, mycobacteriosis, and tularemia were found to be the most common pathogens.^2^^,^^19^ In our study, we have found that dematiaceous fungi, including *Exophila* spp., *Wangiella* spp., *Cladophialophora* spp., *Cladorrhinum bulbillosum*, *Rhinocladiella* spp., *Fonsecace* spp., and unidentified black mold, were the most frequently identified organisms causing sporotrichoid lesions and that the dimorphic *Sporothrix* spp. was an uncommon pathogen. The second most common pathogen was nontuberculous *Mycobacterium*. The pathogens reported in our study were different from those mentioned in previous studies because our study was conducted at a single university-based hospital in southern Thailand where most patients came from metropolitan regions and worked as government officers. In addition, the previous studies were review articles on the online database; further clinical trials are needed to determine accurate results.

Previous studies on cutaneous and subcutaneous infectious granuloma have not focused on the distribution pattern of the lesions, and most studies showed a higher prevalence of mycobacterial infection than of fungal infection.^13^^,^^20^ However, in this study, we observed sporotrichoid distribution of the lesions and a higher prevalence of fungal infection. Thus, in cases of sporotrichoid lymphocutaneous syndrome caused by fungus, antifungal medications should be used for treatment; however, the drug of choice should be determined after the causative pathogen has been confirmed with extensive investigations.

The demographic data of our patients were similar to those reported in other studies: the mean age was nearly 50 years, and female sex was predominant.^11^^,^^12^^,^^16^ The morphologies of the skin lesions varied among the studies. In our study, nodules were the most frequently observed lesions in both fungal and NTM infections, whereas plaques were common in the fungal group but uncommon in the NTM group. In addition, ulcerative lesions were only observed in the fungal group. This finding was similar to that reported in a previous study,^2^ but we found no statistically significant association between skin lesion morphology and causative pathogen. However, previous studies have reported a high frequency of plaques in cases of NTM infections.^12^^,^^13^^,^^21^ Therefore, skin biopsy for tissue culture and histopathological examination must be performed for all skin lesions that are highly suspicious.

Skin trauma was found to be a risk factor for exploring infections.^2^^,^^19^ In the present study, over half of the patients had experienced different types of skin injuries. Mammal-caused injures, such as cat/dog scratch/bite or other exposures to pets, were the most common traumas, followed by thorn prick, aquatic injuries, and insect bites. The geographic location of the study population may have affected the incidence rates of different injuries. Fungal infections seemed to be associated with mammal-caused injuries, but there was no statistically significant association. Although fungal pathogens are mainly found in soil, plants, and organic material, the disease was usually transmitted to humans by infected animals (e.g., cat or dog).^22^ However, in previous studies, nearly half of the study population could not recall the source of their external cutaneous injuries.^13^^,^^23^

The location of infection was found to be correlated with type of injury. In our study, upper extremities were the most common sites of lesions, and this could be explained by the patients being more likely to feed or play with infected pets using their hand; this finding was also reported in previous studies.^6^

Regarding treatment, itraconazole was the most frequently prescribed drug in empirical and antifungal treatment. A previous study also reported that itraconazole has become the treatment of choice for sporotrichosis and dematiaceous fungal infection,^24^ but it may not always be effective in treating the lesions.^14^ However, we recommend using itraconazole as a first-line medication for suspected or identified fungal infection. A recent study of skin infections caused by *M. marinum* in Thailand reported that ciprofloxacin-clarithromycin combination therapy and doxycycline monotherapy were effective regimens.^12^ These findings should be considered when treating outpatients.

### Strengths.

To our knowledge, this is the first study involving patients with sporotrichoid lymphocutaneous infections in Thailand.

### Limitations.

Two-thirds of the patients were excluded for lack of skin biopsy results; this may have led to participant bias. Due to the retrospective nature of the study, clinical data were not collected. In addition, we excluded patients who missed follow-up appointments; moreover, the incidence of the disease has been increasing in the last 2 years, and this period was not included in this study. Therefore, the incidence of sporotrichoid cutaneous syndrome may have been underreported. Considering the high frequency of culture-negative results, some pathogens may not have been identified. The molecular diagnostic technique would greatly improve the positive rate of pathogen detection. However, molecular diagnostic testing remains unavailable in our setting.

## CONCLUSION

Dematiaceous fungi are the most frequently identified pathogens that cause sporotrichoid lymphocutaneous syndrome in southern Thailand. The patients usually present with lesions showing linear distribution along the lymph tract. Empirical treatment with itraconazole should be started especially for those who report pet-related injury at the primary lesion site. Skin biopsy for histopathological examination and culture are essential to perform in suspicious cases.

## Supplemental files


Supplemental materials
