# Supplementary material for: Epidemiological, Clinical, and Histopathological Characteristics of Patients with Sporotrichoid Lymphocutaneous Infection in Southern Thailand: A 10-Year Retrospective Study
Source: Am J Trop Med Hyg. 2023 Feb 13;108(4):696–700. doi: 10.4269/ajtmh.22-0300 (PMC10076999; doi:10.4269/ajtmh.22-0300)
Supplement: Supplementary file 1 [file tpmd220300.SD1.pdf]

Supplementary materials

Table S1. Skin morphologies regarding to pathogens

	Exophiala spp.	*Wangiella* spp.	*Cladorrhinum bulbillosum*	*Rhinocladiella* spp.	*Fonsecaea* spp.	*Sporotrix* spp.	Unidentified black mold	NTM
Nodule	5	3	1	1	0	1	2	3
Papule	1	3	0	0	0	0	1	1
Plaque	3	1	0	0	1	1	0	1
Patch	0	0	0	0	0	0	0	0
Pustule/ Abscess	1	0	0	0	0	0	2	0
Ulcer	5	1	1	0	0	0	0	0

Table S2. Histopathologic features of cutaneous infection with sporotrichoid pattern

Histopathology	Number of patients
**First Biopsy**	**53**
Supparative granuloma	16
Tuberculoid granuloma	5
Mixed granuloma	1
Unspecified granuloma	23
Abscess/no granuloma	4
Unknown/inadequate	1
**Re biopsy**	8
**Reason of re biopsy**	
Inadequate sample	1
Failed empirical treatment	6
Relapsed lesion	1
**Re biopsy results**	
Newly histopathology findings	4
Newly identified pathogens	1

Table S3. Identified pathogens in patient with cutaneous infection with sporotrichoid pattern

Pathogens	Number of patients
**Positive culture**	**24 (45.3%)**
**Fungus**	19 (79.2%)
Exophila spp.	7 (29.1%)
Wangiella spp.	6 (25.0%)
Cladorrhinum bulbillosum	1 (4.2%)
Rhinocladiella spp.	1 (4.2%)
Fonsecaea spp.	1 (4.2%)
Unidentified black mold	2 (8.3%)
Sporothrix spp.	1 (4.2%)
**Nontuberculous mycobacterium**	5 (20.8%)
Mycobacterium marinum	2 (8.3%)
Mycobacterium scrofulaceum	2 (8.3%)
Mycobacterium abscessuss	1 (4.2%)
**Negative culture**	**29 (54.7%)**
